# Fingerprint Database Reconstruction Based on Robust PCA for Indoor Localization

**DOI:** 10.3390/s19112537

**Published:** 2019-06-03

**Authors:** Lingwen Zhang, Teng Tan, Yafan Gong, Wenkao Yang

**Affiliations:** School of Electronic and Information Engineering, Beijing Jiaotong University, Beijing 100044, China; zhanglw@bjtu.edu.cn (L.Z.); 18120056@bjtu.edu.cn (Y.G.); wkyang@bjtu.edu.cn (W.Y.)

**Keywords:** indoor localization, RSS, outlier, low rank, Robust PCA

## Abstract

The indoor localization method based on the Received Signal Strength (RSS) fingerprint is widely used for its high positioning accuracy and low cost. However, the propagation behavior of radio signals in an indoor environment is complicated and always leads to the existence of outliers and noises that deviate from a normal RSS value in the database. The fingerprint database containing outliers and noises will severely degrade the performance of an indoor localization system. In this paper, an approach to reconstruct the fingerprint database is proposed with the purpose of mitigating the influences of outliers. More specifically, by exploiting the spatial and temporal correlations of RSS data, the database can be transformed into a low-rank matrix. Therefore, the RPCA (Robust Principle Component Analysis) technique can be applied to recover the low-rank matrix from a noisy matrix. In addition, we propose an improved RPCA model which takes advantage of the prior knowledge of a singular value and could remove outliers and structured noise simultaneously. The experimental results show that the proposed method can eliminate outliers and structured noise efficiently.

## 1. Introduction

Recently, with the rapidly increasing location-based service (LBS) [1], such as positioning, tracking, navigation, and location-based security, the indoor localization has attracted wide attention. In outdoor environments, Global Positioning Service (GPS) [2] and other satellite positioning methods can provide people with an acceptable accuracy of positioning services, which can basically meet the various needs of people. However, for indoor scenes, the GPS method cannot work due to a signal’s dispersion and blocking. Thus, various indoor localization technologies have been proposed by researchers like Time Of Arrival (TOA) [3], Angle Of Arrival (AOA) [4], Time Difference of Arrival (TDOA) [5], and Received Signal Strength (RSS) [6]. Concerning factors such as positioning accuracy, technical complexity, electromagnetic interference, and construction cost, the WLAN (Wireless Local Area Networks) indoor positioning technology based on RSS fingerprints has become the principal method with an acceptable positioning accuracy.

The fingerprint-based method is widely used in a RADAR system [7], a Nibble system [8], and a Weyes system [9] currently. Generally, the fingerprint-based positioning method is implemented in two phases: the off-line training phase and the online positioning phase. In the off-line training phase, the RSSs from different access point (APs) are measured at the selected reference points (RPs). The RSS information and the corresponding locations are generally formulated as the fingerprint database, which infers the relationship between the RSS distribution and the spatial locations. In the online positioning phase, the RSS information collected in real time is matched to the fingerprint database and the location can be estimated by many proposed methods, such as k-nearest neighbor algorithm (KNN) [7], the kernel-based algorithm [10], a Support Vector Machine (SVM) [11], and the Bayesian estimation method [12].

The fingerprint database is vital for the RSS-based method. However, some studies show that the signal propagation suffers from several noisy characteristics such as interference, reflection, refraction, and even temperature or humidity [13,14] which cause incorrect data in fingerprint database. Moreover, there are numerous causes of outliers [15] in the fingerprint database, for instance, the severe environment factors like human walking, furniture movement, or wall blocking and hardware factors such as incorrect hardware configuration, hardware malfunction, or malicious attacks. The noise and outliers will affect the accuracy of the fingerprint database and result in incorrect positioning information in the online phase.

### 1.1. Related Work

We briefly summarize the relevant studies in this section.

#### 1.1.1. Outlier Suppression Preprocessing

**Figure 1:**
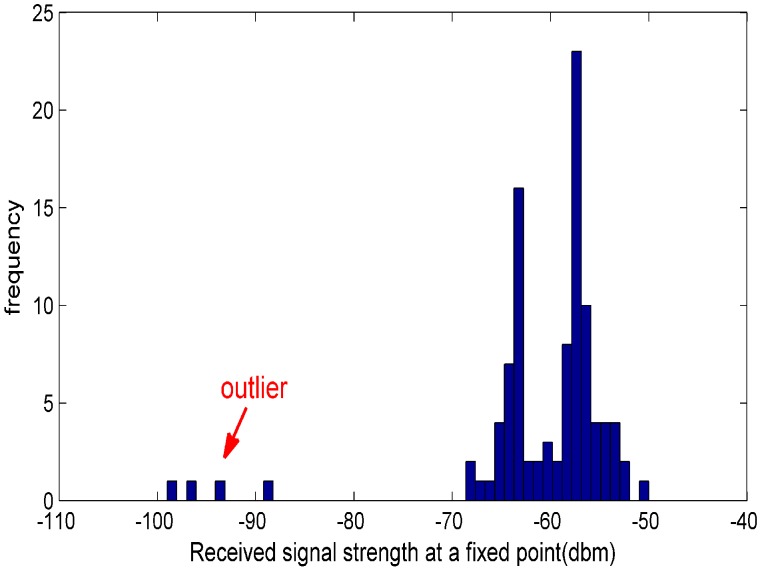
A typical statistical view for an outlier.

Statistically, outliers are the inconsistent points with respect to the normal data in the data set and will destroy the intrinsic structure of the data set. A typical statistical view of outliers in RSS measurements is shown in Figure 1, which was collected in a real indoor environment at a fixed reference point at different times. All these outliers existing in a fingerprint database will degrade the localization accuracy significantly.

**Figure 2:**
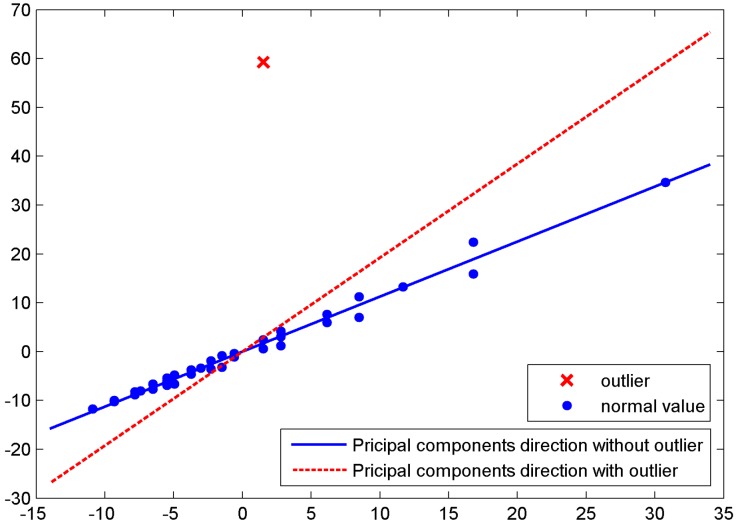
The outlier influence on the principal direction in a standard Principal Component Analysis (PCA).

Therefore, after constructing the fingerprint database in the off-line phase, a preprocessing step is needed to deal with the outliers and noise. Otherwise, outliers will lead to wrong feature extraction and affect the estimation in the online localization algorithm such as SVM or KNN technology. As we can see in Figure 2, when outliers exist, the main direction of projection after the Principal Component Analysis (PCA) process has deviated from the correct line seriously, so it is vital to detect and eliminate noise and outliers.

There are many outlier suppression methods in indoor localization; the median filter [16] is the most popular and simplest way to deal with the RSS variation. Chen et al. [17] presented a method which combines the hampel filter and the kernel density estimator (KDE) by assigning each data a confidence indicator and by judging whether it is credible. Fang et al. [18] presented a new approach to improve the positioning accuracy based on the Singular Value Decomposition (SVD) noise reduction technique.

There are also many outlier detection methods in the data analysis field. Knorr et al. [19] proposed an outlier detection algorithm, which effectively solved the problem of unknown dataset distribution, but it was time-consuming. Ramaswamy et al. [20] presented a k-nearest neighbor outlier detection algorithm based on distance, which determined a point as an outlier if it was far from most of the points. Ester et al. [21] proposed a new clustering algorithm DBSCAN (Density-Based Spatial Clustering of Applications with Noise) that relies on a density-based notion of clusters which was designed to discover clusters of arbitrary shape. Raymond et al. [22] proposed an outlier detection algorithm based on clustering, which detected outliers by examining the relationship between objects and clusters. Markus et al. [23] presented a new outlier detection approach based on the density of data by giving each object a degree called LOF (Local Outlier Factor) for being an outlier. Raymond et al. [22] developed a new clustering method to detect outliers called CLARANS (Clustering Large Applications Based Upon Randomized Search) which was based on a randomized search. Papadimitrio et al. [24] proposed an outlier detection algorithm by introducing MDEF (multi-granularity DEviation Factor), which degraded the computation complexity, but it was hard to select an appropriate parameter.

These traditional dectection methods can accurately find outliers. However, they cannot deal with outliers well by only simply eliminating outliers or by replacing outliers with sample mean values. Therefore, we introduce the RPCA approach, which will be explained in the following section.

#### 1.1.2. Robust PCA

Candès et al. proposed a problem called RPCA [25] to separate a low-rank matrix and a sparse matrix from a sum matrix. The RPCA problem was transformed into a convex optimization problem under minimal assumptions, which was also called Principal Component Pursuit (PCP). For the PCP problem, Candès proposed an iterative thresholding (IT) [25] algorithm which was simple and convergent, but its convergence rate was slow. Lin et al. [26] proposed an accelerated proximal gradient (APG) algorithm, which was several orders of magnitude faster than the IT algorithm. Ma et al. [27] applied the method of Augmented Lagrange Multipliers (ALM) to solve the PCP problem, which achieved a higher accuracy and required less memory space. For RPCA research, many researchers proposed their own improvements. Sun et al. [28] presented a novel non-convex formulation using the capped trace norm and the capped $\ell_{1}$-norm and presented two algorithms to solve the non-convex optimization. Candès et al. [29] proposed a weighted formulation of $\ell_{1}$-norm minimization, which was designed to penalize nonzero coefficients more equally. Rao et al. [30] proposed a new model for robust sparse and low-rank decompositions by introducing the $\ell_{1/2}$-norm for matrices to induce their lower rank. Oreifej et al. [31] proposed a three-term decomposition for video stabilization and moving-object detection, including the low-rank matrix corresponding to the background, the sparse matrix corresponding to the moving object, and the dense error matrix. Xu et al. [32] proposed an RPCA via outlier pursuit to obtain a robust decomposition when the outliers corrupted entire columns and identified the corrupted columns. Kang et al. [33] proposed a novel non-convex rank approximation function that was tighter than the nuclear norm in PCP. Chiang et al. [34] studied RPCA with a consideration for side information and explored the prior structure and entry features for recovery. For the application of RPCA technology, Wright et al. [25] applied RPCA to remove shadows from face images and to subtract video backgrounds. Additionally, RPCA has been successfully applied to image denosing, face modeling, image alignment, and so on.

Based on these researches, taking into account the mixed noise including outliers and structured noise and exploiting the prior knowledge of singular value, we propose an improved RPCA model by introducing $\ell_{2,1}$-norm and by weighting each singular value which will be presented in Section 3.

### 1.2. Motivation and Contribution

Through the above analysis, we can see that a user may get wrong positioning information when the fingerprint database contains noise and outliers. These problems motivated us to propose a method to remove outliers and to purify the database. We noticed that the RSS data of each adjacent measurement has a high spatial correlation and that RSSs collected at a fixed reference point from an access point at different times also show high temporal correlations. The high correlation performs as a low-rank property in a matrix. Therefore, we construct a merging matrix which merges and arranges all the RSS values collected at different locations and different times and combine it with a low-rank recovery theory to remove noise and outliers. The major contributions of this paper can be listed as follows:

- We propose a fingerprint database reconstruction framework based on RPCA and present an improved weighted nuclear norm and multi-norm RPCA model, which utilizes the prior knowledge of singular values to enhance the low-rank property and eliminates both the outliers and structured noise in the meantime.
- We employ the well-known Augmented Lagrangian Multiplier (ALM) method to design a useful algorithm for the proposed model. In the meantime, aimed at the problem of the inability of the fixed direction of the Alternating Direction Method of Multipliers (ADMM) to accomplish convergence, we introduce the Randomly Permuted ADMM method.
- We analyze various different ways to construct a merging matrix to find the optimal matrix to leverage the hidden structure and redundancy of the collected data. The experiments confirm that our approach achieves the best performance, outperforming the other methods.

### 1.3. Organization

The remainder of this paper is organized as follows. The background knowledge and system model for fingerprint-based localization are introduced in Section 2. The framework for reducing outliers and noise in fingerprint database and an improved RPCA model for fingerprint database reconstruction are illustrated in Section 3. In Section 4, the corresponding optimization algorithm for the improved RPCA model is proposed. Some comparison experiment results are evaluated and discussed in Section 5. The conclusion is given in Section 6.

## 2. Fingerprint-Based Localization System

**Figure 3:**
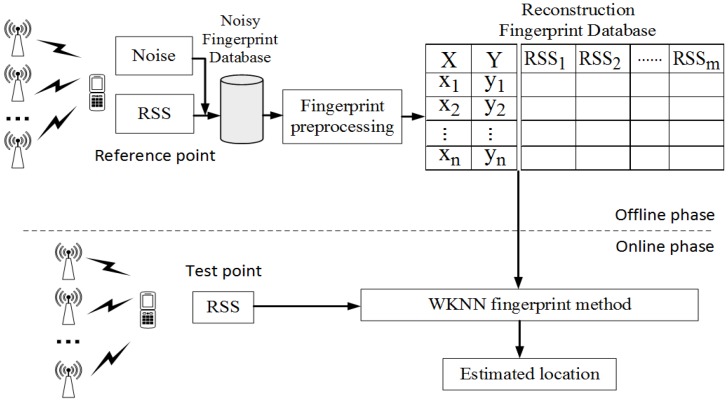
System model.

A typical Wi-Fi indoor positioning system involves an off-line phase and an online phase. In the off-line phase, the main purpose is to construct a fingerprint database which indicates the correspondence between RSSs and reference points’ locations. After collecting real-time RSS in the online phase, the users’ locations will be estimated by using some localization algorithms. The system model is shown in Figure 3.

### 2.1. Off-Line Phase: Fingerprint Database Construction

In the off-line phase, firstly, the reference points should be carefully selected and recorded with their location coordinates. Then, the collected RSSs at the reference points and the corresponding location coordinates constitute the fingerprint database. Consequently, in the fingerprint database, there are two spaces mapped to each other, which are the position location space and the RSS space. Assuming that there are *n* RPs and *m* APs and that $L\in R{}^{n\times 2}$ contains location coordinates of reference points,

(1) $$L={\left[\left(x_{1},y_{1}\right),\left(x_{2},y_{2}\right),\cdots ,\left(x_{n},y_{n}\right)\right]}^{T}$$

where ${\left[\cdot \right]}^{T}$ represents the matrix transpose and x,y represent the *x* coordinate and the *y* coordinate of the reference points, respectively.

Let $\Psi \in R{}^{m\times n}$ be the RSSs collected at the reference points:

(2) $$\Psi =\left[\begin{array}{cccc} \psi_{11} & \psi_{12} & \cdots & \psi_{1n}\\ \psi_{21} & \psi_{22} & \cdots & \psi_{2n}\\ \vdots & \vdots & \vdots & \vdots \\ \psi_{m1} & \psi_{m2} & \cdots & \psi_{mn} \end{array}\right]$$

where $\psi_{ij}$ represents the RSS collected at the *i*th reference point from the *j*th AP. Then, the fingerprint database is expressed as follows:

(3) FingerprintDatabase=[L,Ψ]

In fact, in the practical application scenario, it is inevitable to mix nonstationary noise with the RSS space during the period of fingerprint database constructing, due to human walking, terminal diversity, indoor channel environment change, and other factors. Thus, its vital to have a preprocessing part to keep the RSS space away from the noise and outliers after constructing the fingerprint database.

### 2.2. Online Phase: Localization Algorithm Design

In the online phase, the user terminal receives RSS information $\hat{\psi }\in R{}^{m\times 1}$ at a real-time location and matches it with the RSS space Ψ in the fingerprint database to estimate the user’s location from the known reference points’ locations L. There are many proposed localization algorithms like Weighted KNN (WKNN), SVM, and Bayesian estimation. WKNN is a popular algorithm which is improved from KNN technology due to its computation simplicity and high estimation accuracy. The estimation of the WKNN algorithm is based on the Euclidean distance:

(4) $$d_{j}={∥\psi_{j}-\hat{\psi }∥}_{2}\;\;\;\;\;\;\;\;\;\forall j=1,\cdots ,m$$

where ${∥\cdot ∥}_{2}$ is the $\ell_{2}$-norm operator, $d_{j}$ is the Euclidean distance, and $\psi_{j}$ is the column of Ψ.

In the WKNN algorithm, the distance values are given weight:

(5) $$w_{j}=1/\left(d_{j}+\delta \right)$$

where δ is a small positive number introduced in order to control the denominator as not being zero and δ=0.001 in this paper and where *j* is the index of the reference points obeying 1≤j≤m.

Then, choose k(k>1) reference points which have the shortest Euclidean $d_{j}$ as the candidate positions, and the user’s position is estimated by averaging all *k* candidate positions as follows:

(6) $$\left(\hat{x},\hat{y}\right)=\frac{\sum_{j=1}^{k}w_{j}\left(x_{j},y_{j}\right)}{\sum_{j=1}^{k}w_{j}}$$

In conclusion, we could see that the fingerprint database plays a crucial role in the fingerprint-based indoor localization system. If the RSS space Ψ is not accurate or faults are in the database, the estimated position results would definitely be poor. Therefore, we need a precise fingerprint database to provide a better position estimation.

## 3. Proposed Fingerprint Database Reconstruction Framework

Based on the system model in Section 2, the fingerprint database could be disturbed by outliers. In view of this problem, exploiting the low-rank property of the fingerprint database, we propose a fingerprint database reconstruction framework based on RPCA. Additionally, taking into account the physical meaning of a singular value and the mixed noise situation including outliers and structured noises, we propose an improve RPCA model by weighting nuclear norm and by joining the $\ell_{2,1}$-norm.

### 3.1. The Low-Rank Property Analysis of the Fingerprint Database

#### 3.1.1. The Spatial Correlation of RSS Data

Before solving these problems, we notice that the RSS data has high spatial and temporal correlations. Suppose a fingerprint database Ψ is made up of an RSS vector $\varphi_{i}$ received at an *i*th reference point, that is, $\Psi =[\varphi_{1},\varphi_{2},\cdots ,\varphi_{n}]$. As we know, signal strength measurement relies on the characteristic of the signal propagation. Due to path loss, the signal strength attenuates depending on the distance that the signal travelled. Therefore, the reference points i,j in proximity to one another which have similar distances should have similar RSS feature vectors $\varphi_{i},\varphi_{j}$, which means that the degrees of freedom of the fingerprint database are much lower than the dimensions. The limited degrees of freedom in a matrix shows the spatial correlation of the fingerprint database and exhibits a low-rank property.

**Figure 4:**
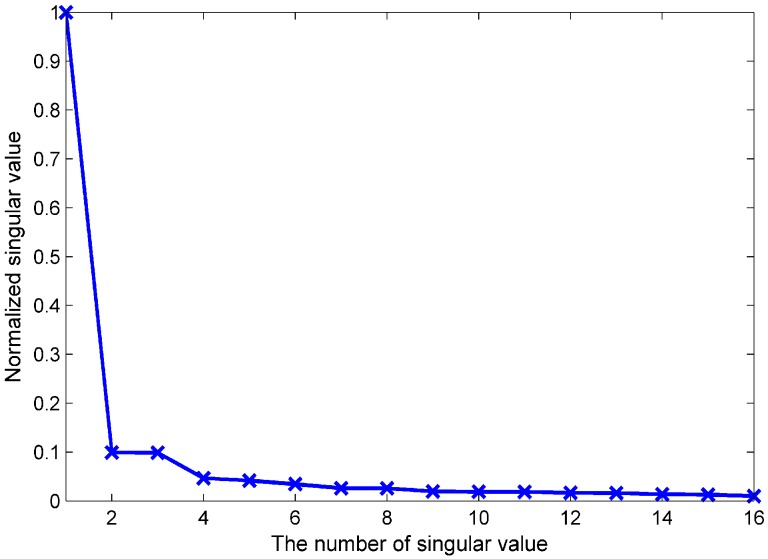
The normalized singular values of the fingerprint database.

To prove the spatial correlation, we construct an example of the fingerprint database Ψ by a logarithmic path loss model. By setting 16 APs and 49 RPs, we can get a fingerprint database $\Psi \in R^{16\times 49}$. Using SVD decomposition technology, the normalized singular value is shown in Figure 4. As we can see, the first singular $\sigma_{1}$ occupies the vast majority of energy and the rest of the singular values $\left(\sigma_{2},\cdots ,\sigma_{16}\right)$ approach zero. As the rank of the matrix is equal to the number of the singular values, we can see the spatial correlation and low-rank property of the fingerprint database.

#### 3.1.2. The Temporal Correlation of RSS Data

Except the spatial correlation in the fingerprint database, RSS values collected from an AP at a fixed location at different times are similar, which reveals the temporal correlation of RSS. Existing work [35] has proved the temporal correlation of RSS. It identified that the RSS values from an AP at a fixed position are highly autocorrelated and shows that the autocorrelation of consecutive samples is as high as 0.9. This high autocorrelation exhibits that, over a short period of time, the signal strength received from an access point at a particular location is relatively stable.

Suppose that $\Psi_{w}$ is the fingerprint database constructed in the *w*th time measurement. Based on the temporal correlation of the RSS data, the fingerprint database $\Psi_{1},\Psi_{2},\cdots ,\Psi_{W}$ constructed at the first, second, and *W*th times are correlated. By merging the fingerprint database constructed at different times into a matrix, the temporal correlation will generate a redundancy in the matrix and the matrix will exhibit a low-rank property eventually.

Therefore, exploiting the spatial correlation and temporal correlation of the fingerprint database constructed at different time measurements, we can form a merging matrix D by merging and arranging all the RSS values collected from *M* APs at *N* different locations at *W* different times. The spatial and temporal correlations of fingerprints result in a low rank of the merging matrix. Let D be the merging matrix of fingerprints with dimensions $\Psi \in R^{W\cdot M\times N}$:

(7) $$D=[\Psi_{1};\Psi_{2};\cdots ;\Psi_{W}]$$

where $\Psi_{w}$ represents the fingerprint database constructed in the *w*th (w=1,2,⋯,W) time as the sub-matrix and $\Psi_{w}\left(m,n\right)$ is the RSS collected from the *m* th (m=1,2,⋯M) AP at the *n*th (n=1,2,⋯N) location. The merging matrix will be the low-rank matrix in the subsequent RPCA processing.

### 3.2. Strategy on Organizing the Matrix

In the previous subsection, the size of the merging matrix is W·M×N, where W,M,N refer to the collecting times, the amount of APs, and the number of RPs, repectively, and we set them to 9, 36, and 5, respectively. However, this is not the only way to construct the merging matrix. Different structures of a matrix make different contributions to its inner correlations, which will further influence the effectiveness of RPCA. Hence, it is necessary to find a suitable matrix that can be recovered most effectively.

We list all possible combinations of matrices in Table 1 and give a new meaning to each matrix. The value of *W* represents the number of block matrix, and the sign of *W* indicates the orientation of the block matrix, where “+” represents the block matrix arrayed in rows and “−” represents the block matrix arrayed in columns. M,N represent the number of rows and columns of the matrix. For instance, if W·M×N is +5·36×9, then $X=[\Psi_{1},\Psi_{2},\cdots ,\Psi_{5}]$, where $\Psi_{i}$ is a 36×9 block matrix.

**Figure 5:**
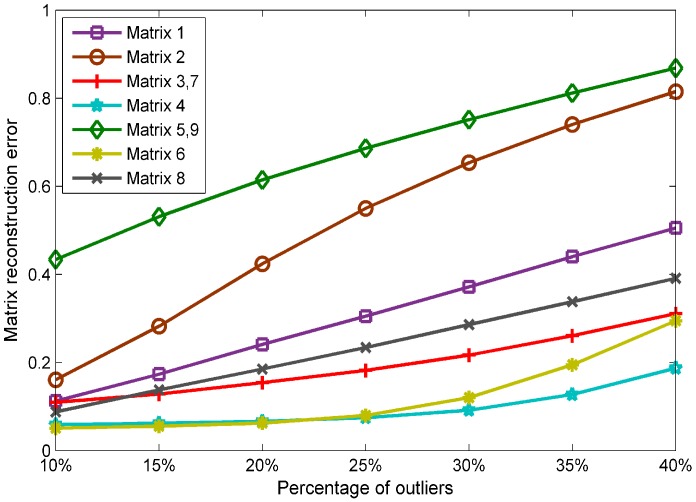
The matrix reconstruction error with different structures.

Figure 5 shows the performance with different sizes of merging matrix in a matrix reconstruction error, where the *x*-axis represents the percentage of outliers and the *y*-axis represents the matrix reconstruction error. As we can see, the matrix performance in different structures shows great differences; thus, it is vital to select the final size of the merging matrix. Through the analysis of Figure 5, we choose the matrix with size of 45×36 in the following simulation experiments.

### 3.3. Fingerprint Database Reconstruction Based on RPCA

In this section, we exploit the low-rank property of the merging matrix to fix the outlier problem hidden in the fingerprint database and establish corresponding mathematical models. Notice that Candès presented a framework called RPCA to solve the following decomposed problem: Suppose there is a data matrix D, which is the sum of the low-rank matrix A and the sparse matrix E; how can we decompose the low-rank and sparse components accurately and efficiently?

Wright et al. [36] have proved that, when $D\in R^{n_{1}\times n_{2}}$, $n=\max(n_{1},n_{2})$ and $m=\min(n_{1},n_{2})$, the decomposition succeeds with a probability at least $1-cn^{-10}$, provided that

(8) $$rank\left(A\right)\leq \rho_{r}m\mu^{-1}{\left(\log n\right)}^{-2}\;\;\;\mathrm{and}\;\;\;\;x\leq \rho_{s}n_{1}n_{2}$$

where μ, $\rho_{r}$, *c*, and $\rho_{s}$ are positive numerical constants; *x* is the number of corrupted entries; and $\lambda =1/\sqrt{n}$.

The merging matrix constructed by RSS has a low-rank property which is demonstrated in the previous subsection. Therefore, based on the low-rank recover theory, we can remove the noise and outliers contained in the fingerprint database. Assuming that the merging matrix D contains noise and outliers, matrix A is the precise matrix after denosing that we need online positioning and matrix E is the sparse matrix which represents the noise and outliers. Thus, the mathematical optimization model for separating outliers from the fingerprint database can be built as follows:

(9) $$\begin{array}{cc} \min & \mathrm{rank}(A)+\lambda {∥E∥}_{o}\\ s.t. & D=A+E \end{array}$$

where the constraint ${∥\cdot ∥}_{o}$ represents the number that is the nonzero element in the matrix, forcing *E* to be sparse, and the parameter λ>0 controls the trade-off between two terms.

However, the problem in Equation (9) is generally an NP-hard problem. Thus, Candès transformed it into a convex optimization problem by relaxing the non-convex rank function as a nuclear norm ${∥\cdot ∥}_{*}$ and by relaxing ${∥\cdot ∥}_{0}$ as ${∥\cdot ∥}_{1}$. The transformed problem is formulated as follows:

(10) $$\begin{array}{c} \min\;\;\;\;\;{∥A∥}_{*}+\lambda {∥E∥}_{1}\\ s.t.\;\;\;\;\;\;\;\;\;\;D=A+E \end{array}$$

where ${∥A∥}_{*}$ denotes the nuclear norm of the matrix A, that is, the sum of the singular value $\sigma_{i}\left(A\right)$ and ${∥E∥}_{1}$ denotes the $\ell_{1}$-norm of the matrix E.

By solving this optimization problem, we can recover the accurate fingerprint database A with no noise and outliers from the original noisy fingerprint database D.

### 3.4. An Improved RPCA Optimization Model

In this section, we propose an improved RPCA model by taking into account the mixed noise including outliers and structured noise and by exploiting the prior knowledge of a singular value to propose an improved RPCA model.

#### 3.4.1. Join $\ell_{2,1}$-Norm as Structured Noise

In practical applications, the fingerprint database may contain structured noise, which represents that a few columns or rows in the matrix are completely corrupted when some situations occur; for instance, APs blocked by obstacles or an AP hardware malfunction such as a low battery will result in an unstable transmitted power.

Noticing that structured noise is not taken into account in the standard RPCA model [25], we introduce a new matrix G to represent the structure noise. By using $\ell_{2,1}$-norm to capture the corruption structure, the improved RPCA mathematical model is constructed as follows:

(11) $$\begin{array}{c} \min\;\;\;\;\;{∥A∥}_{*}+\lambda {∥E∥}_{1}+\gamma {∥G∥}_{2,1}\\ s.t.\;\;\;\;\;\;\;\;\;\;D=A+E+G \end{array}$$

where the ${∥G∥}_{2,1}$ denotes the $\ell_{2,1}$-norm, that is, ${∥G∥}_{2,1}=\sum_{i}{\left(\sum_{j}G_{ij}^{2}\right)}^{\frac{1}{2}}$, the i,j denotes the index of the rows and columns, and the parameter γ represents the trade-off between the matrix decomposed.

#### 3.4.2. Weighted Nuclear Norm

Furthermore, notice that the nuclear norm ${∥\cdot ∥}_{*}$, which is the sum of the singular values $\sigma_{i}$, treats each singular value equally. As a result, the soft thresholding operator shrinks each singular value with the identical value. However, it ignores the prior knowledge that the large singular values occupy the principal components of the matrix, which should be shrunk less, while the small singular values may contain noise or redundant components, which should be shrunk larger.

Therefore, we take different weights for each singular values to improve the flexibility of the nuclear norm and construct a new optimization model as follows:

(12) $$\begin{array}{c} \min\;\;\;\;\;{∥A∥}_{w,*}+\lambda {∥E∥}_{1}+\gamma {∥G∥}_{2,1}\\ s.t.\;\;\;\;\;\;\;\;\;\;D=A+E+G \end{array}$$

where ${∥A∥}_{w,*}$ denotes the weighted nuclear norm, that is, ${∥X∥}_{w,*}=\sum_{i}\left|w_{i}\sigma_{i}(X)\right|$ and $w=[w_{1},\cdots ,w_{n}]$, where $w_{i}>0$ is a nonnegative weight assigned to $\sigma_{i}(X)$.

In this way, we construct the improved RPCA model as Equation (12). By solving this optimization problem, we can decompose the matrix E which represents outliers and matrix G which represents structured noise from the measurement matrix D and get the low-rank matrix A as the accurate fingerprint database. Additionally, we design an efficient algorithm for the improved RPCA model using the well-known ALM method and will illustrate it in detail in the next section.

## 4. Algorithm Derivation

In this section, the ALM method is adopted to solve the proposed model, which solves the constrained optimization by transforming it into an unconstrained optimization problem. To solve the proposed optimization problem by using ALM, we have use the preliminary definitions and theorems as follows:

### 4.1. Preliminary Definition

#### 4.1.1. Definition 1

Shrinkage Operator: For any τ>0 and $X\in R^{m\times n}$, the shrinkage operator $S_{\tau }\left(X\right)$ is defined as

(13) $$S_{\tau }\left(X_{ij}\right)=\left\{\begin{array}{c} X_{ij}-\tau \;\;\;\;\;x>\tau \\ X_{ij}+\tau \;\;\;\;\;x<-\tau \\ 0\;\;\;\;\;otherwise \end{array}\right.$$

#### 4.1.2. Definition 2

Soft-thresholding Operator: For any τ>0 and $X\in R^{m\times n}$ with a Singular Value Decomposition $X=U\sum V^{T}$, the soft-thresholding operator is

(14) $$D_{\tau }\left(X\right)=US_{\tau }\left(\sum \right)V^{T}$$

#### 4.1.3. Theorem 1

For any τ>0 and $X\in R^{m\times n}$, the Shrinkage Operator is the optimal solution of the function as

(15) $$S_{\tau }\left(X\right)=\arg\min_{X}\left\{\frac{1}{2}{∥X-Y∥}_{F}^{2}+\tau {∥X∥}_{1}\right\}$$

#### 4.1.4. Theorem 2

For any τ>0 and $X\in R^{m\times n}$, the Soft-thresholding Operator is the optimal solution of the function as

(16) $$D_{\tau }\left(X\right)=\arg\min_{X}\left\{\frac{1}{2}{∥X-Y∥}_{F}^{2}+\tau {∥X∥}_{*}\right\}$$

#### 4.1.5. Theorem 3

For any τ>0 and $X\in R^{m\times n}$, the $\hat{X}$ is the optimal solution of the function of

(17) $$\min\frac{1}{2}{∥X-Y∥}_{F}^{2}+\eta {∥X∥}_{2,1}$$

and the *i*th row of $\hat{X}$ is

(18) $$\hat{X}\left(i,:\right)=J_{\eta }\left(X\right)=\left\{\begin{array}{c} \frac{{∥Y_{i}∥}_{2}-\eta }{{∥Y_{i}∥}_{2}}Y_{i},\;\;\;\;\;\;if{∥Y_{i}∥}_{2}>\eta \;\\ 0,\;\;\;\;\;\;\;\;\;\;\;\;\;\;\;\;\;\;\;\;\;\;\;\;\;\;\;\;\;\;\;\;\;\;\;\;\;\;\;\;\;\;\;\;\;otherwise\; \end{array}\right.$$

### 4.2. Transform to Unconstrained Problem by ALM

To solve the optimization problem, we firstly convert the constrained optimization problem into an unconstrained optimization problem by introducing a Lagrangian multiplier Y and a quadratic penalty term and then formulate the augmented Lagrange function as follows:

(19) $$\begin{array}{c} \begin{array}{c} L\left(A,E,Y,\mu \right)={∥A∥}_{*}+\lambda {∥E∥}_{1}+\gamma {∥G∥}_{2,1}+<Y,D-A-E-G>+\frac{\mu }{2}{∥D-A-E-G∥}_{F}^{2} \end{array} \end{array}$$

where the operator <·> represents the inner product of two matrices, ${∥\cdot ∥}_{F}$ is the Frobenius norm which represents tolerable errors, matrix Y is the Lagrange multiplier matrix, and μ>0 is the tunable parameter.

### 4.3. Iteration Steps

After the transformation into an unconstrained problem, the Alternating Direction Method of Multipliers is adopted to solve the proposed Lagrangian function of Equation (19). However, the fixed-direction alternating iteration cannot achieve convergence efficiently; thus, we utilize the randomly permuted ADMM, which randomly selects the direction of iterations for each variable in each iteration step. By regarding the other variable as a constant term in each iteration, we have the following iterative steps:

Step 1: update A

(20) $$\begin{array}{cc} A_{k+1} & =\arg\min_{A}L\left(A,E_{k},G_{k},Y_{k},\mu_{k}\right)\\ & =\arg\min_{A}{∥A∥}_{*}+\frac{\mu }{2}{∥D-A-E-G+\mu^{-1}Y∥}_{F}^{2}\\ & =D_{w/\mu }\left(D-E_{k}-G_{k}+\mu^{-1}Y\right) \end{array}$$

Step 2: update E

(21) $$\begin{array}{cc} E_{k+1} & =\arg\min_{E}L\left(A_{k+1},E,G_{k},Y_{k},\mu_{k}\right)\\ & =\arg\min_{E}\lambda {∥E∥}_{1}+\frac{\mu }{2}{∥D-A-E-G+\mu^{-1}Y∥}_{F}^{2}\\ & =S_{\lambda /\mu }\left(D-A_{k+1}-G_{k}+\mu^{-1}Y\right) \end{array}$$

Step 3: update G

(22) $$\begin{array}{cc} G_{k+1} & =\arg\min_{E}L\left(A_{k+1},E_{k+1},G,Y_{k},\mu_{k}\right)\\ & =\arg\min_{E}\eta {∥G∥}_{2,1}+\frac{\mu }{2}{∥D-A-E-G+\mu^{-1}Y∥}_{F}^{2}\\ & =J_{\eta /\mu }\left(D-A_{k+1}-E_{k+1}+\mu^{-1}Y\right) \end{array}$$

Step 4: update Y

(23) $$Y_{k+1}=Y_{k}+\mu_{k}\left(D-A_{k+1}-E_{k+1}-G_{k+1}\right)$$

Then, update the parameter $\mu_{k+1}=\rho \mu_{k}$ in order to accelerate the iteration. The iteration stops until it satisfies the criterion ${∥D-A_{k+1}-E_{k+1}-G_{k+1}∥}_{F}/{∥D∥}_{F}<\varepsilon$, where the ρ and ε are nonnegative constants. Following the above steps, the whole optimization procedure for the improved RPCA model is summarized in Algorithm 1.

**Algorithm 1** Proposed Weighted Nuclear Norm and Multi-Norm RPCA Algorithm**Input:** merging matrix D, tolerance ε, Maximum Convergence Number Ω, parameters λ, η**Output:** low rank matrix A, sparse matrix E, structure matrix G, number of iterations τ.
1:Initialize parameter ρ,μ, A=0, E=0, G=0, Y.2:**while** not converged **do**3: Randomly choose direction to iterate *A*, *E* and *G*;4: When solve $A_{k+1}=\arg\min_{A}L\left(A,E_{k},Y_{k},\mu_{k}\right)$:;5: $\left(U,S,V\right)=svd\left(D-E_{k}+{\mu_{k}}^{-1}Y_{k}\right)$.;6: $A_{k+1}=US_{w/\mu }\left(S\right)V^{T}$;7: When solve $E_{k+1}=\arg\min_{E}L\left(A_{k+1},E,Y_{k},\mu_{k}\right)$:8: $E_{k+1}=S_{\lambda /\mu }\left(D-A+1/\mu Y\right)$;9: When solve $G_{k+1}=\arg\min_{E}L\left(A_{k+1},E_{k+1},G,Y_{k},\mu_{k}\right)$:10: $G_{k+1}={}_{\eta /\mu }\left(D-A_{k+1}-E_{k+1}+\mu^{-1}Y\right)$;11: Update $Y_{k+1}=Y_{k}+\mu_{k}\left(D-A_{k+1}-E_{k+1}\right)$;12: Update $\mu_{k}$ to $\mu_{k+1}$;13: k→k+1.14:
**end while**
15:
**return** $A_{k},E_{k},G_{k}$



## 5. Numerical Experiments

To evaluate the proposed method, we implement the fingerprint database reconstruction experiments on both simulated and real data. The performance can be evaluated by the following three indicators: the average positioning error, matrix reconstructed error, and structured noise recognition accuracy. The average positioning error is defined as

(24) $$localization\_error=\sqrt{{\left(\overset{\wedge }{p}\left(x\right)-p\left(x\right)\right)}^{2}+{\left(\overset{\wedge }{p}\left(y\right)-p\left(y\right)\right)}^{2}}$$

where $\overset{\wedge }{p}\left(x\right)$ and $\overset{\wedge }{p}\left(y\right)$ represent the *x* and *y* coordinates of the estimated position and where p(x) and p(y) represent the *x* and *y* coordinates of real position.

The matrix reconstructed error is defined as

(25) $$matrix\_error=\frac{{∥\hat{X}-X∥}_{F}}{{∥X∥}_{F}}$$

where the ${∥\cdot ∥}_{F}$ denotes the Frobenius norm, X is an ideal matrix without noise, and $\hat{X}$ is the reconstructed matrix.

The structured noise rows recognition accuracy is defined as

(26) $$r=2\cdot \frac{r_{{}_{1}}\cdot r_{2}}{r_{1}+r_{2}},r_{1}=\frac{r_{true}}{r_{all}},r_{2}=\frac{r_{true}}{r_{act}}$$

where $r_{true}$ and $r_{all}$ denote the number of structured noise recognized by the proposed algorithm and the number of the true structured noise among them, respectively, and $r_{act}$ is the actual number of rows of structured noise.

### 5.1. Simulation Experiments

To simulate the RSS in indoor localization and to test the performance of each algorithm, we use the ray-tracing method to simulate the signal attenuation. The simulation field is 20×20×4 m, which contains 9 APs and 36 RPs. They contain seven ray paths, including the direct path, the reflection paths of the four walls, the ground, and the ceiling in an indoor environment. The electric intensity of each path is generated as follows:

(27) $$E_{i}=R_{i}\cdot \frac{\lambda }{4\pi d_{i}},i=\left(1,2,\cdots ,7\right)$$

where λ is the wavelength of electromagnetic wave, $d_{i}$ is the travel distance of the *i*th path, and $R_{i}$ is the reflection coefficient. The dielectric constant are set to 5+0.1j for walls and 6−1.2j for the ground and the ceiling.

The received signal strength in each reference point could be calculated as follows:

(28) $$P_{r}=P_{t}+2G_{l}+20\log_{10}\left(\left|\sum_{i=1}^{7}E_{i}\right|\right)$$

where $P_{t}$ is the transmitted power which we set to 15 dbm and $G_{l}$ is the Antenna gain which we set to 2.15 dbm.

After preprocessing the fingerprint database in the off-line training phase, we apply the WKNN technology to the online positioning phase for a better presentation of performance. In the online positioning phase, we randomly select 1000 test points in the simulation field as users to test the localization performance.

Three categories of contrast experiments are designed to evaluate the performance under different noise conditions, i.e., the outlier, structured noise, and mixed noise including outliers and structured noise. The proposed algorithm is compared with four methods, i.e., IALM (Inexact Augmented Lagrange Multipliers) algorithm, which is the best algorithm for the traditional RPCA model so far; the DB(pct,d) method, which is a classical distance-based outlier detection algorithm; and the KNN method, which detects outliers based on the neighbors’ distances and the Hampel filter method. Taking into account the randomness of the size and location of the noise, all the results of the experiment are the average of 500 independent repetitive experiments. In the meantime, considering the randomness of noise, we have done simulation experiments in different percentages and values of noise. Three contrast experiments are listed as follows:

- WONS: with outlier noise, no structured noise.
- NOWS: no outlier noise, with structured noise.
- WOWS: with outlier noise, with structured noise.

#### 5.1.1. Experiment Results under WONS

Experiments in this set assume that the fingerprint database is contaminated by outliers, which is generated randomly in different ranges, added at random positions, and obeys uniform distribution and that gain or loss are also randomly generated. To test the five methods, we use the Kruskal–Wallis test by setting an α level equal to 0.05 with a sample size of $n_{i}=20\;\left(i=1,2,3,4,5\right)$. We establish a null hypothesis H0—the five methods do not have significant differences in the mean localization error—and the alternative hypothesis H1—the five methods have differences for the same percentage of outliers which is set to 5%. By calculating the revised statistics $H_{c}=73.532$, which is greater than the critical value 9.488 when the degree of freedom v=4 and the significance value α=0.05, so H0 is rejected and H1 is accepted. Therefore, we can conclude that the five processing methods are statistically different.

**Figure 6:**
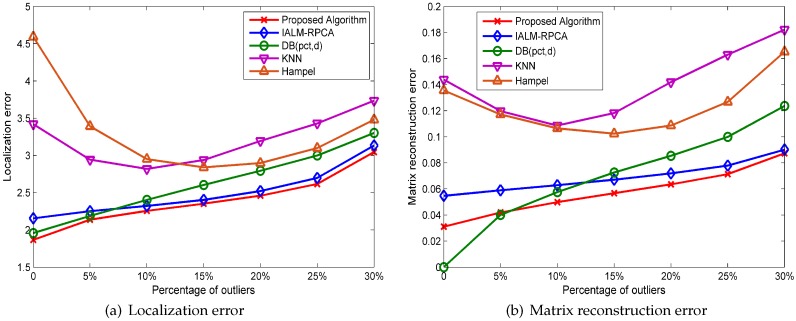
The performance evaluation at different percentages of outliers under the WONS (with outlier noise, no structured noise) condition.

As we can see in Figure 6a, where the *x*-axis represents the percentage of outliers added in the merging matrix and the *y*-axis represents the average localization error, while outliers are generated randomly ranging from 50 to 100, the RPCA-based methods perform better than the outlier detection method when the percentage of outliers is more than 10%. Additionally, the KNN and Hampel methods have poor performances when the percentage of outliers is small, and the proposed algorithm has the best performance in localization error in all cases. Figure 6b shows the matrix reconstruction error in different percentage of outliers. DB(pct,d) has the best performance when the percentage of outliers is zero, which shows that this method can distinguish outliers and normal points accurately, but the RPCA-based method has a better performance than DB(pct,d) when the percentage of outliers is more than 10%. Additionally, the proposed algorithm has the best performance in matrix reconstruction error compared with others when the percentage of outliers is more than 5%.

**Figure 7:**
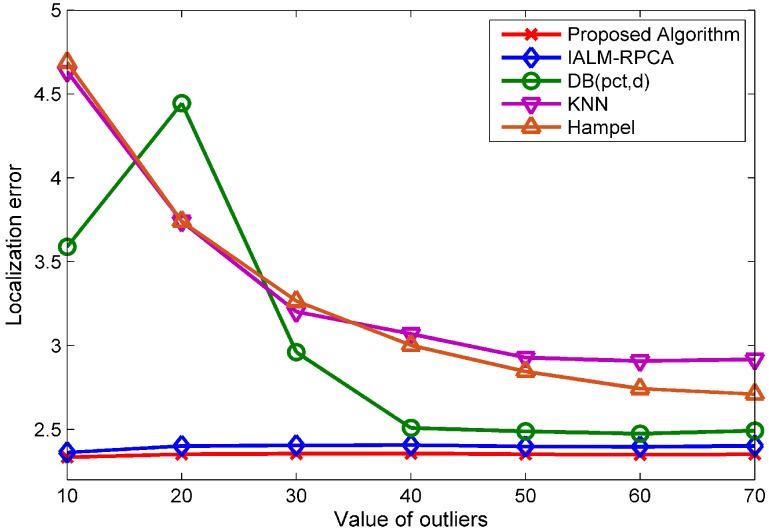
The localization error for different values of outliers under WONS.

Figure 7 shows the mean localization error of the five methods when the value of outliers changes, which means that the outliers are generated randomly, ranging from the value to double the value; for instance, if the value of outliers is 10, the outliers are generated randomly, ranging from 10 to 20, and the percentage of outliers is set to 10%. We can see that the localization error changes slightly when the value of outliers changes for the RPCA-based method while the outlier detection method cannot handle the outliers well when the value of outliers is small and that the proposed algorithm has the lowest localization error compared with the other methods in all cases.

#### 5.1.2. Experiment Results under NOWS

Experiments in this section assume that the fingerprint database is affected by the structured noise which is added in random rows. Each element value of structured noise is generated randomly from −15 to 15. We apply the Kruskal–Wallis test to the five methods by setting an α level equal to 0.05 with a sample size of $n_{i}=20\;\left(i=1,2,3,4,5\right)$ and establish a null hypothesis H0—the median of the localization error after the five methods’ processing do not have significant differences—and the alternative hypothesis H1—the five methods are not exactly the same for the same percentage of structured noise, which is set to 10%—by calculating the revised statistics $H_{c}=84.835$, which is greater than the critical value 9.488 when the degree of freedom v=4 and the significance value α=0.05. Therefore, H0 is rejected and H1 is accepted, and we can conclude that there are differences between the five methods in a statistical sense.

**Figure 8:**
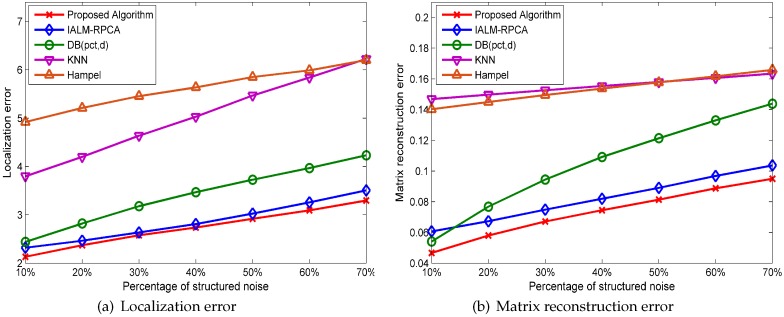
The performance evaluation in different percentages of structured noise under the NOWS (no outlier noise, with structured noise) condition.

Figure 8a illustrates the five methods’ performances in localization error, where the *x*-axis represents the percentage of structured noise rows and the *y*-axis represents the mean localization error. We can see that the performance of the proposed algorithm and IALM preform better than the outlier detection method in all cases and that the proposed algorithm has the best performance and the lowest localization error compared with the other methods. The matrix reconstruction error in a different percentage of structured noise is shown in Figure 8b. As we can see, the RPCA-based method has the lowest matrix reconstruction error and DB(pct,d) has a good performance when the percentage of structured noise is small, while the KNN and Hampel methods cannot handle the structured noise‘well.

**Figure 9:**
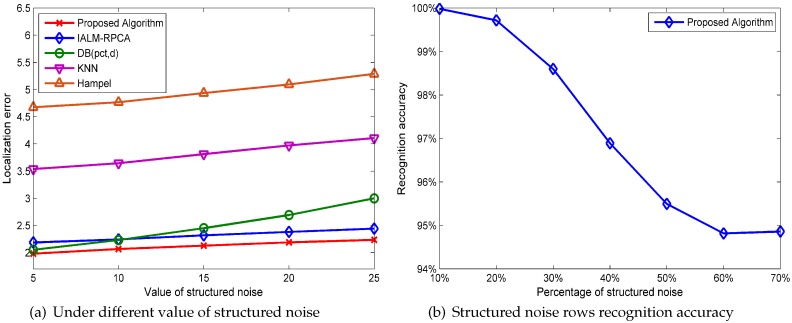
The performance evaluation under NOWS.

Figure 9a shows the localization error of the five methods for different values of structured noise when the percentage of structured noise is set to 10%. The value *p* of structured noise means that each element of structured noise is generated randomly form the −p to *p*, for instance, the value of the structured noise generated randomly from −5 to 5 when the value is 5. As we can see, the KNN and Hampel methods have poor performances under NOWS while the proposed algorithm has the best performance compared with the other methods in all cases.

Furthermore, we can find that our algorithm is competent in recognizing the structural noise positions, i.e., the out algorithm can estimate the node of AP which was broken down and unable to provide effective information. The recognition accuracy performance is showed in Figure 9b, where the *x*-axis represents the percentage of structured noise and the *y*-axis represents the accuracy of detecting structured noise. We can see that the accuracy to find the right position of the structured noise has a probability of 100% when the percentage of structured noise is 10% and also has more than a 94% accuracy in the other cases. According to this characteristic, we can eliminate the failure AP which provides wrong information in an online localization.

#### 5.1.3. Experiment Results under WOWS

Experiments in this part assume that the fingerprint database is contaminated by the mixed noise including outlier noise and structured noise. Outliers generated randomly in different ranges obey uniform distribution, and structured noise is generated randomly from −15 to 15. We apply the Kruskal–Wallis test to the five methods by setting an α level equal to 0.05 with a sample size of $n_{i}=20\;\left(i=1,2,3,4,5\right)$. We establish a null hypothesis H0—the mean localization error after three methods’ processing do not have significant differences—and the alternative hypothesis H1—the three methods have differences in localization error for the same percentage of mixed noise including 5% outliers and 10% structured noise. We calculate the statistics $H_{c}=80.538$, which is greater than the critical value 9.488 when the degree of freedom v=4 and the significance value α=0.05, so H0 is rejected and H1 is accepted. Thus, we can conclude that there are differences between the five processing methods.

**Figure 10:**
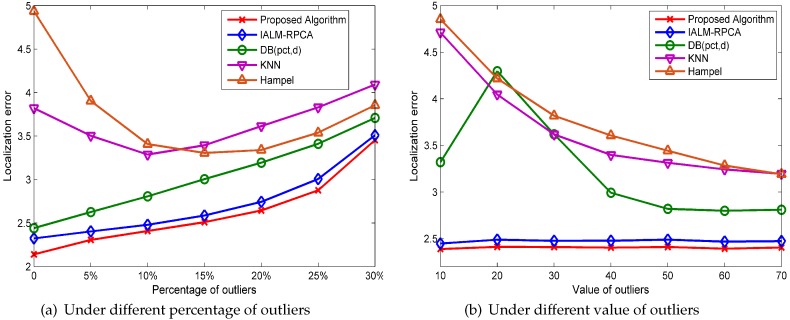
The performance evaluation under the WOWS (with outlier noise, with structured noise) condition.

Figure 10a shows the localization performance under different percentages of outliers, while the value of the outliers is generated randomly, ranging from 50 to 100, and the number of structured noise rows is set to 10%. As we can see, the RPCA-based method performs better than the traditional outlier detection method and our algorithm has the best performance when the percentage of outliers is less than 30%.

The performance for different values of outliers is shown in Figure 10b, where the *x*-axis represents the value of outliers and *y*-axis represents the localization error, and the percentage of outliers is set to 10%. RPCA-based methods change slightly when the value of outliers changes, and our algorithm has a better performance than IALM in all cases, while the DB(pct,d), KNN, and Hampel methods cannot handle the situation well when outliers are small.

**Figure 11:**
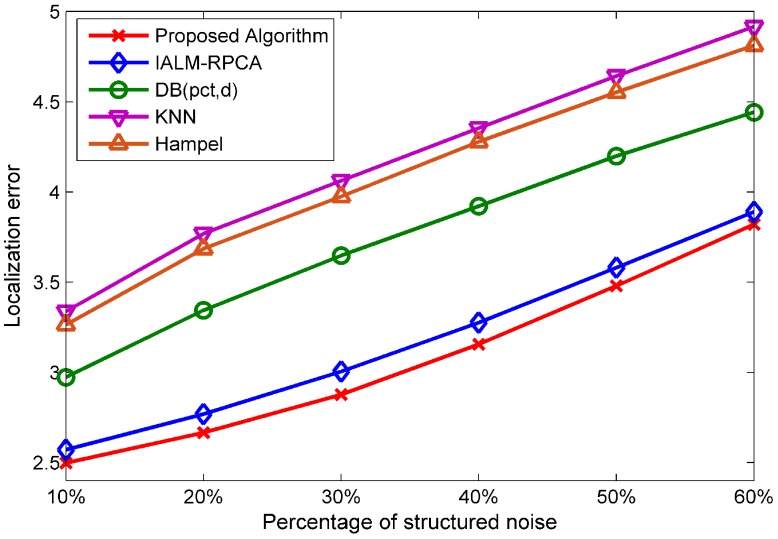
The performance at different percentages of structured noise under WOWS.

Figure 11 shows the performance of the five methods at different percentages of structured noise rows, while the values of the outliers are generated randomly, ranging from 50 to 100. The percentage of outliers is set to 15%, and the structured noise value is generated randomly, ranging from −15 to 15. We can see that our algorithm has the best performance and that the minimum localization error as the number of structured noise increases.

### 5.2. Real-World Experiments

**Figure 12:**
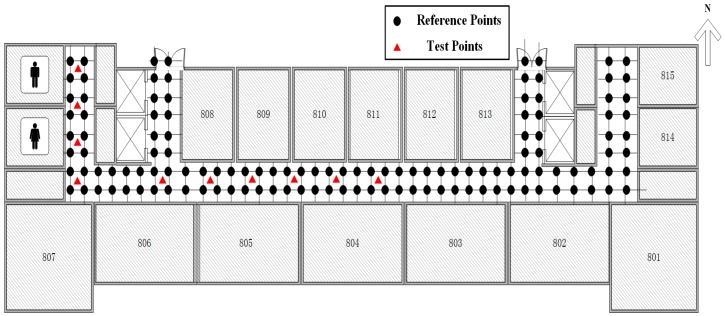
A comparison of different algorithm results in term of Cumulative Distribution Function (CDF).

To test the performance of the proposed algorithms, we performed experiments in a school building. The floor plan is shown in Figure 12. The experiment area includes a long west-east-oriented aisle and four shorter north-south-oriented aisles. The long aisle is around 40 m while the shorter aisle is nearly 8.5 m. There are 16 APs arranged in the area with uniform specifications but unknown positions. The direction from east to west is marked as the *x*-axis. The direction from south to north is marked as the *y*-axis.

The reference points are set symmetrically with a 1.2 m spacing. There are 126 reference points in total, which are represented by dots in Figure 12. In the off-line stage, we use a TL-WN823N USB wireless network adapter which is compatible with the IEEE 802.11 n/g/b standard. The frequency of the system is operated on 2.4 GHz. We record eight times the RSS information at each reference point, and the sampling interval is 1 s.

**Figure 13:**
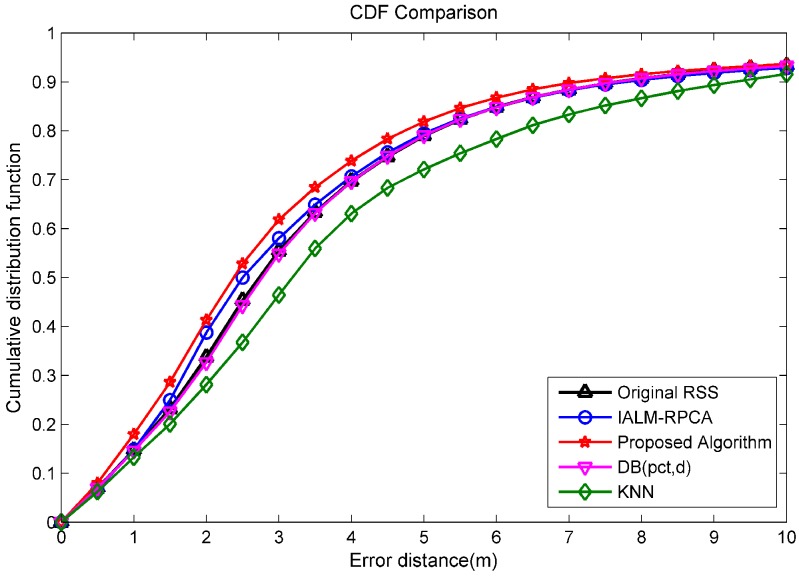
A comparison of different algorithm results in term of CDF.

In the online phase, we selected ten test points which are represented by triangles in Figure 12. In order to have enough data to test the localization performance, we scan the RSS information at every test point 100 times, i.e., 1000 users are considered for localization. The Cumulative Distribution Function (CDF) of the localization error for different methods is shown in Figure 13. There are five methods to process the fingerprint database in Figure 13, including the original RSS from a measurement, the proposed algorithm, the IALM algorithm, the KNN method, and the DB(pct,d) method.

As we can see from Figure 13, the proposed algorithm has the best localization accuracy compared with the other methods and original RSS. Under 4 m, the accuracy of the proposed algorithm is 73%, while the IALM is 70%, and the original RSS is 69%. The DB(pct,d) performs almost the same as the original RSS. Moreover, the KNN method performs even worse than the original RSS, which means that some normal entries are wrongly treated as outliers.

## 6. Conclusions

In this paper, an off-line fingerprint database reconstruction approach is proposed. By observing and proving that the signal has high spatial and temporal correlations, the RSS data can be transformed into a low-rank matrix. Then, the RPCA technique can be applied to recover the matrix without noise. Meanwhile, a novel optimization problem is proposed for simultaneously eliminating the outliers and structured noise. Additionally, a strategy on organizing the low-rank matrix is given. Furthermore, an algorithm is derived to solve the proposed optimization problem based on the ALM method. The superiority of the proposed approach has been demonstrated in several experiments.

## Figures and Tables

**Table 1:** Merging matrices of various sizes.

Index	W	M	N	Final Size
1	+1	6	6	6×6
2	+1	9	36	9×36
3	+1	36	9	36×9
4	−5	9	36	45×36
5	+5	9	36	9×180
6	+5	36	9	36×45
7	−5	36	9	180×9
8	−9	36	5	324×5
9	+9	5	36	5×324
